# Data on the no-load performance analysis of a tomato postharvest storage system

**DOI:** 10.1016/j.dib.2017.06.044

**Published:** 2017-07-01

**Authors:** Orhewere B. Ayomide, Oluseyi O. Ajayi, Solomon O. Banjo, Adesola A. Ajayi

**Affiliations:** a Mechanical Engineering Department, Covenant University, P.M.B. 1023 Ota, Nigeria; b Biological Sciences Department, Covenant University, P.M.B. 1023 Ota, Nigeria

**Keywords:** Energy systems, Heat transfer, Vapour compression refrigeration system, Tomato postharvest storage system, Agriculture engineering, Biosystem engineering

## Abstract

In this present investigation, an original and detailed empirical data on the transfer of heat in a tomato postharvest storage system was presented. No-load tests were performed for a period of 96 h. The heat distribution at different locations, namely the top, middle and bottom of the system was acquired, at a time interval of 30 min for the test period. The humidity inside the system was taken into consideration. Thus, No-load tests with or without introduction of humidity were carried out and data showing the effect of a rise in humidity level, on temperature distribution were acquired. The temperatures at the external mechanical cooling components were acquired and could be used for showing the performance analysis of the storage system.

## Specifications Table

**Table:** 

Subject area	*Mechanical Engineering*
More specific subject area	*Machine design, heat transfer and thermo-fluid engineering*
Type of data	*Table, text file, Graph*
How data was acquired	*The tomato postharvest storage system was designed and constructed using the principle of vapour compression refrigeration system. The external temperatures of the compressor, condenser, evaporator and expansion valve, and the internal temperature distribution of the system׳s chamber were measured with k-type thermocouples and a digital hygrometer was used to measure the humidity in the system. The refrigerant charge and discharge pressures were acquired by a Sigma® testing manifold*
Data format	*Raw, Analyzed*
Data source location	*Department of Mechanical Engineering, Covenant University, Ota Ogun State, Nigeria.*
Experimental factors	*The Walls of the storage system was wet with water to introduce humidity into the system before the No-load, humidity introduction test was performed.*
Experimental features	*No-load tests, were carried out on the designed tomato postharvest storage system for a test period of 96 h, at a 30 min interval. The tests were divided into No-load test with and without humidity introduction. The data acquired were compared to determine the effect on the temperature pull down time at different locations in the storage system, when humidity level is raised.*
Data accessibility	*Data are available within this article*

## Value of the d**ata**

• The given dataset should show researchers the correlation between the external temperatures of the mechanical components (compressor, evaporator, expansion valve and condenser) of the cooling system and this could be used in determining the coefficient of performance and exergy of the system.
• The dataset for the temperature distribution within the system׳s chamber can be employed to determine the air flow pattern, and also determine the impact the system has on the stored tomato. This could further aid in the computational fluid dynamics analysis of temperature distribution within the system׳s chamber.
• The data can be used to investigate the effect of relative humidity on the pull-down time at different locations in the storage system.
• The dataset could be used in investigating the effect of the evaporator position on the warm and cold zone in a system that uses natural convection method of heat transfer.

## 1 Data

The temperatures at the compressor, evaporator, expansion valve and condenser were collected and a set of experimental data was generated. Temperature interaction between the evaporator and air at the top, middle and bottom of the cooling system were collected at 30 min time interval. The No-load experiments were each run twice and the average taken as representative data for better accuracy. Also, data showing the pressure of the refrigerant at the suction and at the discharge of the compressor was gathered (Table 1, Table 2).

**Table 1:** Experimental data showing no-load test, performed on the tomato postharvest storage system.

Time (min)	Average No Load Test										
	Average ambient temperature −29.50 °C										
	*T*1(°C)	*T*2 (°C)	*T*3 (°C)	*T*4 (°C)	*T*5 (°C)	*T*6 (°C)	*T*7 (°C)	*T*8 (°C)	*R*/*H* (%)	*P*1 (psi)	*P*2 (psi)
0	30.00	31.00	29.50	28.00	28.00	29.00	25.50	27.50	79.50	34.00	30.00
30	19.50	53.50	39.00	11.00	14.00	13.50	18.00	15.17	33.00	0.00	92.50
60	17.00	58.50	42.50	10.50	10.00	9.50	15.00	11.50	34.50	0.00	92.50
90	17.00	60.00	42.00	9.50	9.50	7.50	14.00	10.33	36.00	0.00	95.00
120	17.00	61.00	41.50	9.00	9.00	7.00	14.00	10.00	36.50	0.00	95.00
150	15.50	61.50	44.00	10.00	9.00	7.00	14.00	10.00	35.50	0.00	95.00
180	15.50	62.00	44.00	10.00	8.50	7.00	14.00	9.83	36.00	0.00	95.00
210	16.50	62.50	44.00	9.50	8.00	6.50	14.00	9.50	36.00	0.00	95.00
240	16.00	61.50	42.00	9.50	8.50	6.50	14.00	9.67	35.50	0.00	95.00
270	15.50	61.50	42.00	8.50	8.50	6.50	13.50	9.50	35.50	0.00	95.00
300	15.50	60.50	39.50	7.50	7.50	6.00	13.00	8.83	35.50	0.00	95.00
330	16.50	60.00	39.00	7.50	7.50	6.00	13.00	8.83	35.50	0.00	95.00
360	15.50	59.50	40.00	7.50	7.50	6.00	13.00	8.83	36.50	0.00	95.00
390	15.50	58.50	37.00	6.50	7.50	6.00	13.00	8.83	36.00	0.00	95.00
420	15.50	58.50	37.50	7.00	7.50	6.00	13.00	8.83	35.50	0.00	95.00
450	15.00	59.00	39.50	6.50	8.00	6.00	13.00	9.00	35.00	0.00	95.00
480	15.50	58.00	38.00	6.50	7.50	6.00	13.00	8.83	34.50	0.00	95.00
510	15.50	59.50	40.50	7.50	7.50	6.00	13.00	8.83	35.00	0.00	95.00
540	16.50	60.00	40.50	8.00	7.50	6.00	13.00	8.83	35.50	0.00	95.00
570	16.50	60.50	40.50	7.50	7.50	6.50	13.00	9.00	35.00	0.00	95.00
600	15.00	60.50	41.00	8.00	7.50	6.50	13.00	9.00	37.50	0.00	95.00
630	15.00	60.50	40.00	8.00	7.50	6.50	13.00	9.00	37.00	0.00	95.00
660	15.50	58.50	37.50	6.00	7.50	5.50	12.50	8.50	35.50	0.00	95.00
690	16.00	57.00	37.00	6.00	7.50	5.50	12.00	8.33	35.50	0.00	95.00
720	15.50	57.50	37.00	6.00	7.00	5.00	12.00	8.00	34.50	0.00	95.00
750	15.00	56.50	35.50	5.50	7.00	5.50	12.00	8.17	34.00	0.00	95.00
780	14.50	57.50	38.00	6.00	7.00	5.50	12.00	8.17	33.50	0.00	95.00
810	14.50	58.00	38.00	6.50	7.00	5.50	12.00	8.17	33.50	0.00	95.00
840	15.00	58.00	37.00	6.00	7.00	5.50	12.00	8.17	34.00	0.00	95.00
870	14.50	58.00	37.50	6.00	7.00	5.00	12.00	8.00	34.00	0.00	95.00
900	16.00	57.50	36.50	6.00	7.00	5.00	12.00	8.00	34.00	0.00	95.00
930	16.50	57.50	37.50	5.50	6.50	5.00	12.00	7.83	33.50	0.00	95.00
960	16.00	57.50	37.00	5.50	7.00	5.00	12.00	8.00	33.50	0.00	95.00
990	16.50	57.50	36.50	5.50	7.00	5.00	12.00	8.00	33.50	0.00	95.00
1020	16.00	58.00	37.50	6.00	7.00	5.00	12.00	8.00	33.50	0.00	95.00
1050	16.00	57.50	37.00	6.00	6.50	5.00	12.00	7.83	33.00	0.00	95.00
1080	16.00	57.50	36.50	5.50	6.50	5.00	12.00	7.83	33.00	0.00	95.00
1110	16.00	57.50	37.00	6.00	7.00	5.00	12.00	8.00	33.00	0.00	95.00
1140	16.00	58.00	37.50	6.00	7.00	5.50	12.50	8.33	33.00	0.00	95.00
1170	16.00	58.00	37.50	6.00	7.00	5.50	13.00	8.50	33.00	0.00	95.00
1200	16.50	58.00	37.00	6.00	7.00	5.50	13.00	8.50	33.00	0.00	95.00
1230	15.50	58.50	38.00	6.00	7.50	5.50	13.00	8.67	33.00	0.00	95.00
1260	15.50	58.00	37.50	6.00	7.50	5.50	13.00	8.67	33.00	0.00	95.00
1290	15.50	58.00	37.00	6.00	7.50	5.50	13.00	8.67	33.00	0.00	95.00
1320	15.50	58.00	37.50	6.50	7.50	5.50	13.50	8.83	33.00	0.00	95.00
1350	15.50	58.00	38.50	6.50	7.50	6.00	14.00	9.17	33.50	0.00	95.00
1380	15.00	58.50	39.00	6.50	7.50	6.00	14.00	9.17	33.00	0.00	95.00
1410	16.00	59.00	38.50	6.50	8.00	6.00	14.00	9.33	33.50	0.00	95.00
1440	16.50	59.00	38.50	6.50	8.00	6.50	7.00	7.17	33.50	0.00	95.00

**Table 2:** Experimental data showing no-load test when humidity was introduced to the tomato postharvest storage system.

Time (min)	Average No load, Humidity Introduction Test										
	Average ambient temperature −29.5 °C										
	*T*1 (°C)	*T*2 (°C)	*T*3 (°C)	*T*4 (°C)	*T*5 (°C)	*T*6 (°C)	*T*7 (°C)	*T*8 (°C)	*R*/*H* (%)	*P*1 (psi)	*P*2(psi)
0	31.00	32.50	31.00	27.00	28.00	27.50	27.50	27.67	97.50	34.00	30.00
30	21.00	58.00	47.50	17.50	18.00	17.00	25.00	20.00	90.00	3.50	102.50
60	20.50	60.50	45.50	16.00	16.50	14.50	23.50	18.17	94.00	3.00	100.00
90	21.00	61.00	41.50	14.50	15.50	13.00	22.50	17.00	95.00	2.50	97.50
120	19.00	60.50	41.00	14.00	15.00	13.00	21.50	16.50	97.00	0.50	92.50
150	18.50	60.50	40.50	13.00	15.00	12.00	20.50	15.83	95.00	0.00	92.50
180	18.50	59.50	40.00	12.50	14.00	11.50	20.00	15.17	94.50	0.00	90.00
210	19.00	59.50	40.50	12.00	14.00	11.00	19.00	14.67	94.50	0.00	90.00
240	18.00	61.00	44.50	13.00	14.00	11.50	18.50	14.67	94.50	0.00	97.50
270	18.00	61.00	43.00	12.00	14.00	10.50	18.00	14.17	93.50	0.00	95.00
300	18.00	61.00	43.50	12.00	14.00	10.50	17.50	14.00	92.50	0.00	95.00
330	18.00	62.00	45.00	12.50	14.00	10.50	17.50	14.00	92.00	0.00	95.00
360	18.00	61.50	44.50	12.00	13.50	10.50	17.00	13.67	91.00	0.00	92.50
390	17.50	61.50	43.50	11.50	13.00	10.50	16.00	13.17	90.50	0.00	92.50
420	16.50	61.00	44.00	11.00	13.50	10.50	16.00	13.33	89.50	0.00	92.50
450	17.00	60.00	42.00	10.50	13.00	10.00	16.00	13.00	89.00	0.00	92.50
480	16.50	60.00	43.00	11.00	13.00	9.50	15.50	12.67	89.00	0.00	92.50
510	15.50	61.00	42.50	11.50	13.00	9.50	15.00	12.50	88.00	0.00	95.00
540	15.50	61.00	43.00	11.00	13.00	9.50	15.00	12.50	87.50	0.00	95.00
570	15.00	61.00	43.00	11.50	12.50	9.50	15.00	12.33	86.00	0.00	95.00
600	15.50	61.50	41.50	11.00	13.00	9.50	14.50	12.33	86.00	0.00	95.00
630	15.50	62.00	43.00	11.00	13.00	9.50	14.50	12.33	85.00	0.00	95.00
660	15.50	62.00	45.50	11.50	13.00	9.50	14.00	12.17	84.50	0.00	95.00
690	16.50	61.50	38.50	10.50	13.00	9.50	14.00	12.17	84.50	0.00	95.00
720	15.50	63.00	39.50	11.00	13.00	9.50	13.50	12.00	83.50	0.00	95.00
750	15.50	62.00	41.00	11.00	13.00	9.50	13.50	12.00	83.00	0.00	95.00
780	15.50	62.00	42.00	11.00	13.00	9.50	13.50	12.00	82.00	0.00	95.00
810	15.50	61.50	41.50	10.50	12.50	9.50	13.50	11.83	81.00	0.00	95.00
840	15.50	61.50	42.00	10.50	12.50	9.50	13.50	11.83	80.50	0.00	95.00
870	17.50	61.00	42.00	11.00	12.50	9.00	13.50	11.67	81.00	0.00	90.00
900	17.00	61.00	41.50	10.50	12.50	9.00	13.00	11.50	80.00	0.00	92.50
930	16.00	61.50	45.50	11.00	12.50	9.00	13.00	11.50	79.50	0.00	92.50
960	16.00	60.50	42.00	11.00	13.00	9.50	13.00	11.83	79.50	0.00	95.00
990	15.50	60.00	40.00	10.00	12.00	8.50	12.00	10.83	79.00	0.00	95.00
1020	15.00	60.50	42.50	10.50	12.00	8.50	12.00	10.83	79.00	0.00	95.00
1050	15.50	60.00	41.00	10.50	12.50	8.50	12.00	11.00	79.00	0.00	95.00
1080	15.00	60.50	42.50	10.50	12.50	8.50	12.00	11.00	79.50	0.00	95.00
1110	15.00	62.50	46.00	12.00	12.50	9.00	12.00	11.17	80.50	0.00	95.00
1140	17.00	60.00	39.50	9.00	12.50	9.00	12.00	11.17	81.00	0.00	95.00
1170	16.00	60.00	40.00	9.50	13.00	9.00	12.00	11.33	80.50	0.00	95.00
1200	16.00	60.00	39.50	10.00	13.00	9.00	12.00	11.33	80.50	0.00	95.00
1230	14.50	63.00	46.00	12.00	13.50	9.50	12.00	11.67	80.50	0.00	90.00
1260	15.00	63.00	42.00	10.50	13.50	10.00	12.00	11.83	79.50	0.00	95.00
1290	14.00	62.50	41.50	10.00	13.50	9.50	12.00	11.67	80.00	0.00	95.00
1320	13.50	62.00	41.50	10.00	13.00	9.50	12.00	11.50	80.50	0.00	95.00
1350	13.50	61.00	42.00	9.50	13.00	9.50	12.00	11.50	80.00	0.00	95.00
1380	14.00	61.00	42.00	10.00	13.00	9.50	12.00	11.50	80.50	0.00	95.00
1410	13.00	60.50	41.50	9.00	13.00	9.50	12.00	11.50	80.00	0.00	95.00
1440	13.00	60.00	41.00	9.00	13.00	9.00	12.00	11.33	80.00	0.00	95.00

## 2 Experimental design, materials and methods

**Fig. 1:**
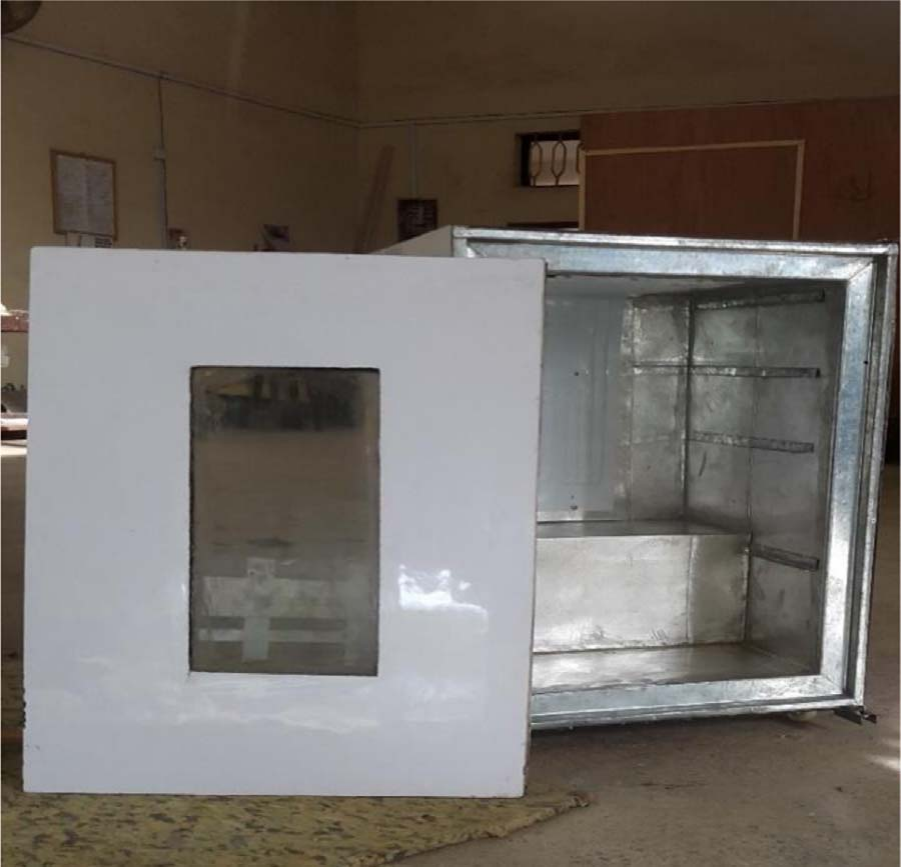
Tomato postharvest storage system.

**Fig. 2:**
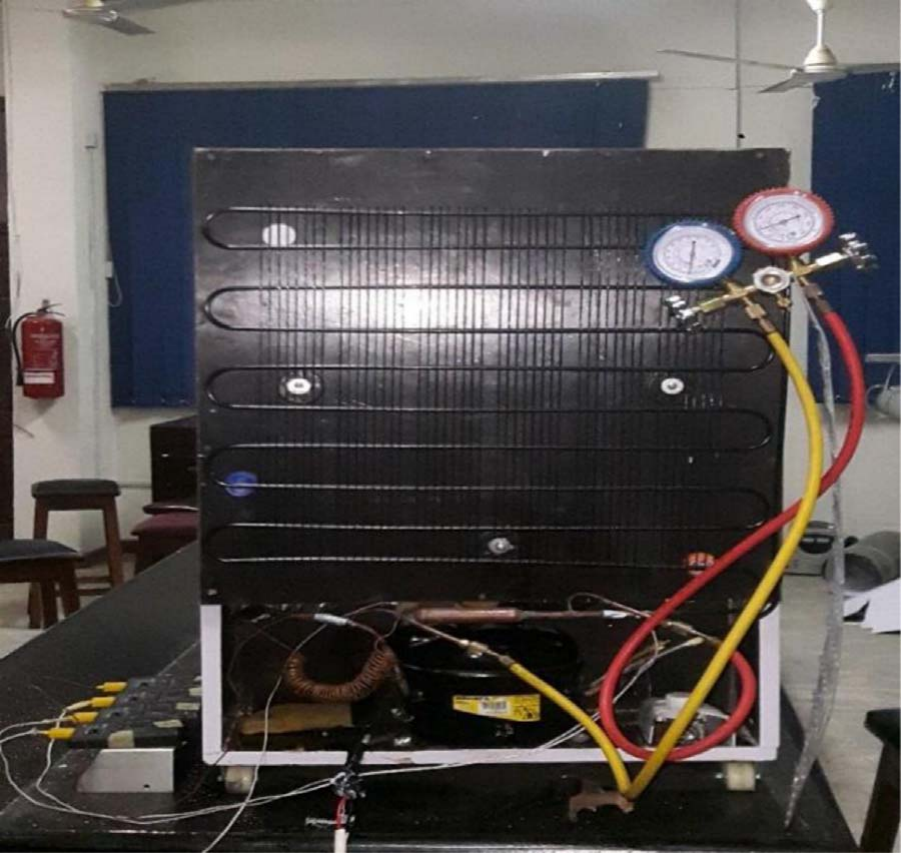
Thermocouples and pressure gauge attached to the external components of the system.

**Fig. 3:**
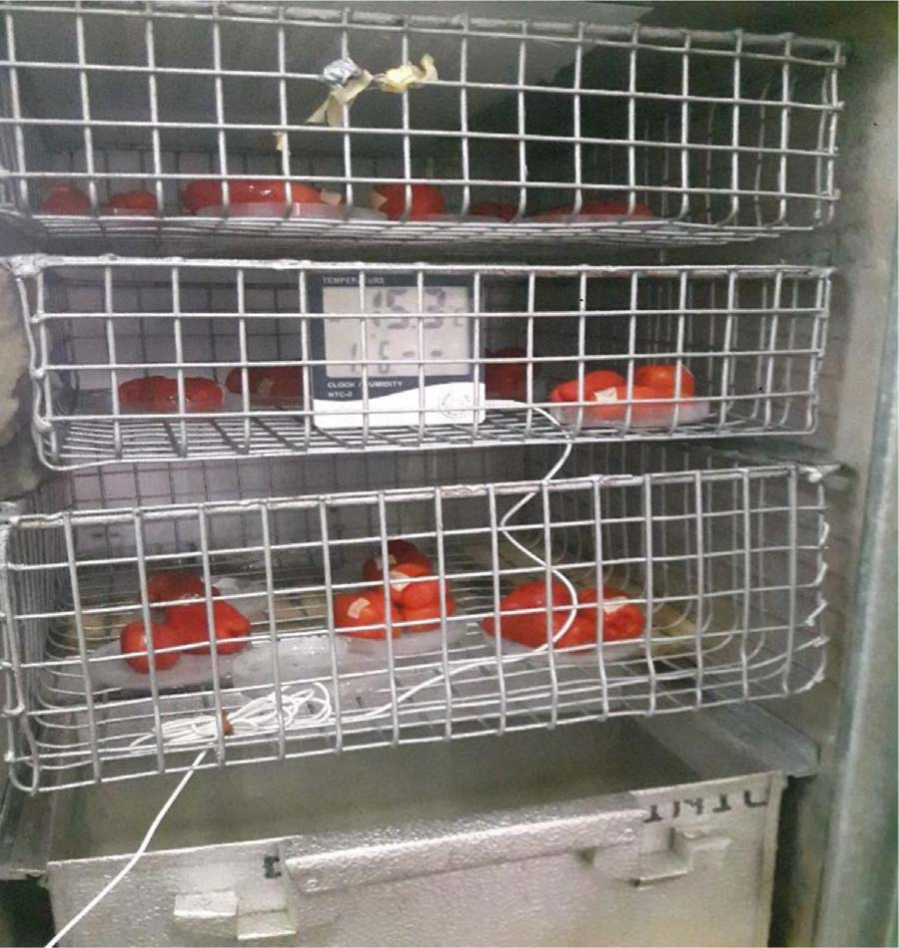
Loaded Tomato postharvest storage system showing the experimental set up.

**Fig. 4:**
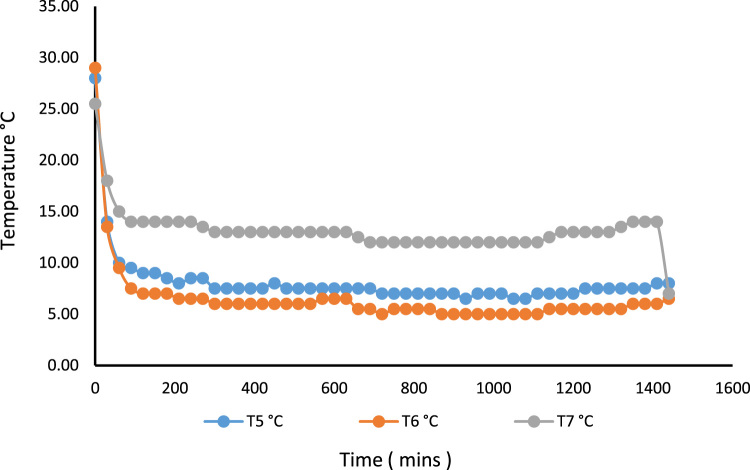
No-Load temperature distribution at three locations within the system, showing the pull down time without humidity introduction.

A 530 mm×560 mm×790 mm (width×depth×height) tomato postharvest storage system was designed and constructed as shown in Fig. 1. The principle of the vapour compression refrigeration system was employed by using a 1/10 aspera R600a compressor, 1/10 air cooled condenser, 1/10 plate and tube evaporator and capillary tube of 0.91mm internal diameter and length 3442 mm, used to control the internal environmental condition of the system. The capacities of the mechanical cooling components were chosen based on the design calculations [1]. The evaporator was positioned within the refrigerating space, from the top to the back of the system. The working fluid responsible for heat exchange process and also known as the life force of a cooling system is the refrigerant [2], [3], [4]. The refrigerant flowing through the system was Isobutane (R600a) charged at a pressure of 2.34 bar. The inner compartment or the cooling chamber was made from aluminum sheet metal. The humidity level in the cooling system was raised by the application of an open 38 cm×20 cm×18.5 cm (width×depth×height) aluminum vessel filled with 14.06 l of water. Data was acquired in form of temperatures at the mechanical cooling components and the internal cooling chamber Fig. 2. Temperatures at the mechanical components can be used in analyzing the coefficient of performance and exergy [5] of the storage system while the internal temperatures of the cooling chamber can be used in analyzing the transfer of heat [6] in the system. Analysis displaying the data are shown in Fig. 1, Fig. 2, Fig. 3, Fig. 4.

*T*_1_=Temperature at the compressor suction (inlet)
*T*_2_=Temperature at the compressor discharge (outlet)
*T*_3_=Temperature at the condenser
*T*_4_=Temperature at the capillary tube/ throttling valve
*T*_5_=Temperature at the top of the cooling chamber
*T*_6_=Temperature at the middle of the refrigerating space
*T*_7_=Temperature at the bottom of the cooling chamber
*T*_8_=Average temperature inside the cooling chamber=$\left\{\frac{T5+T6+T7}{3}\right\}$
*P*_1_=Pressure at the suction (inlet of compressor)
*P*_2_=Pressure at the discharge (outlet of compressor)

**Fig. 5:**
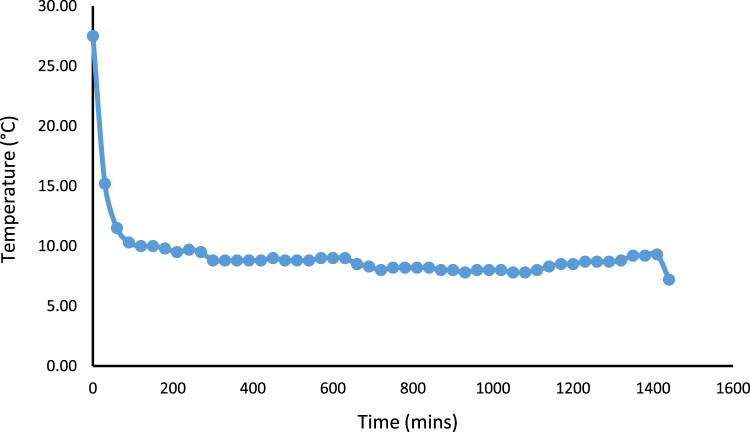
Average temperature distribution across the storage system showing the pull down time without humidity introduction.

**Fig. 6:**
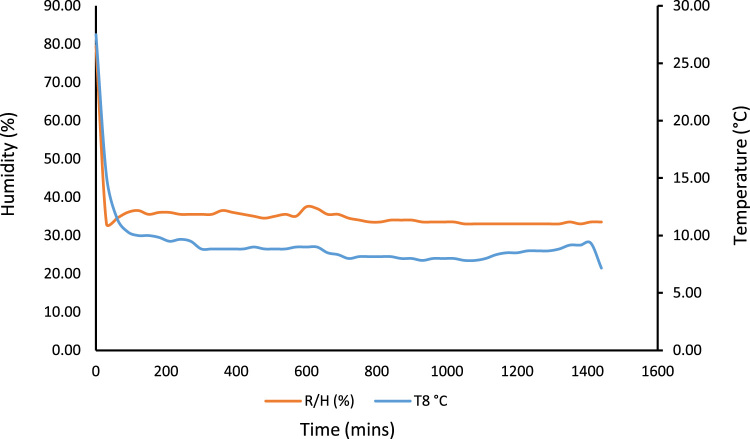
No-Load Test showing the rate of temperature drop pertaining to humidity level in the storage system.

**Fig. 7:**
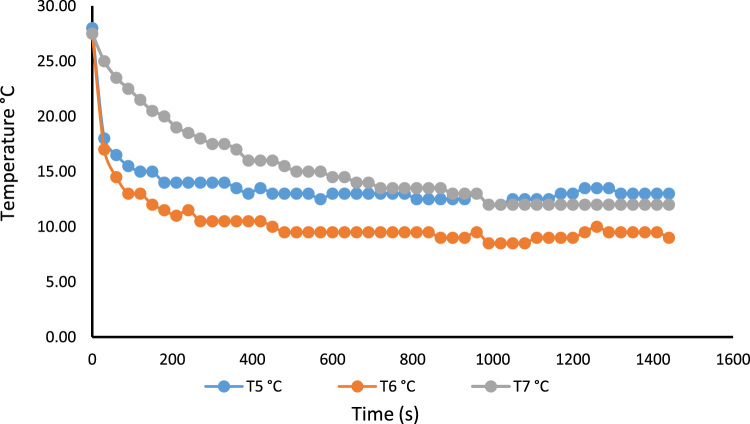
No load temperature distribution at three locations within the storage system showing the pull down time after the introduction of humidity.

**Fig. 8:**
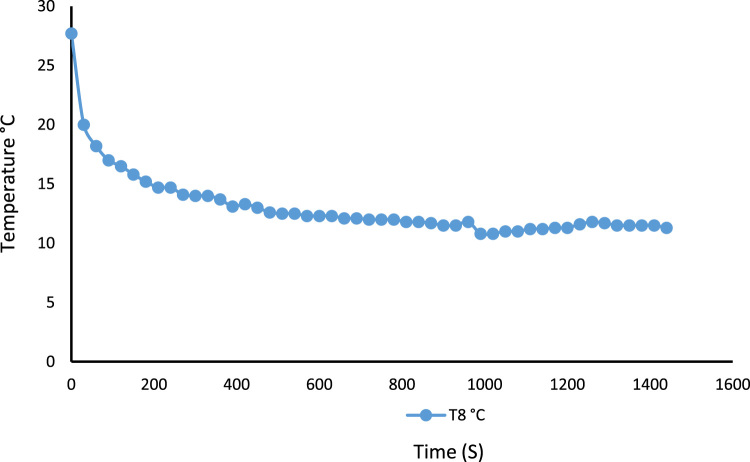
Average temperature distribution across the storage system showing the pull down time after the introduction of humidity.

**Fig. 9:**
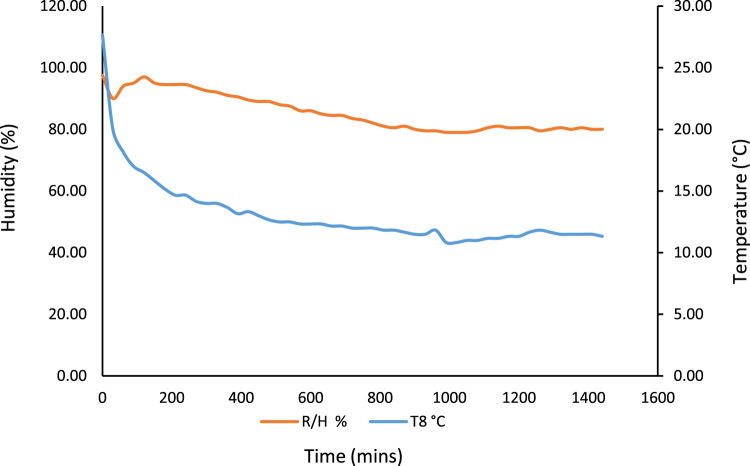
No-Load Test showing the rate of temperature drop pertaining to humidity introduction.
